# A Multi-Indicator Concentration Index for Primary-Care Reimbursement Analytics: Evidence from the Albanian Health-Insurance System

**DOI:** 10.3390/healthcare14172836

**Published:** 2026-09-03

**Authors:** Tomi Thomo, Gjergji Koja, Bujar Elezi

**Affiliations:** 1 Albanian Ministry of Health, 1001 Tirana, Albania; 2 Department of Management and Marketing, Faculty of Economics, Business and Development, European University of Tirana, 1023 Tirana, Albania; 3 Faculty of Medical Sciences, University of Elbasan “Aleksandër Xhuvani”, 3001 Elbasan, Albania; gjergji.koja@uniel.edu.al; 4 Tirana Regional Health Insurance Fund, 1019 Tirana, Albania; bujar.elezi@fsdksh.gov.al

**Keywords:** prescribing variation, primary care, reimbursement screening, outlier detection, peer comparison, healthcare informatics, provider profiling

## Abstract

**Background:** Public payers must regularly decide which prescribers to look at more closely, with records covering thousands of doctors per quarter and review capacity for only a fraction. How that choice is made bears on equitable access to reimbursed medicines as well as on public funds. **Methods:** We propose a multi-indicator concentration index scoring each prescriber 0–100 as the sum of nine weighted sub-scores, each measuring the doctor’s deviation from their primary-care centre median on one indicator from the payer’s routine quarterly report; eight are computable from a single quarter. Every score decomposes into named components. The index measures the concentration of indicators above the peer profile, not an estimated probability of misconduct. It is a screening tool for prioritising documentary review. We demonstrate it on one quarter of anonymised data from 335 doctors in 30 Albanian primary-care centres. **Results:** The eight active sub-scores are mostly weakly correlated (mean |r|=0.28), the two exceptions each pairing a case count with its financial effect. Rankings are robust to alternative weights (Spearman ρ between 0.93 and 0.98), to collapsing those pairs (ρ≥0.98), and to pooling the smallest centres against a shared comparator (ρ=0.95). Against three machine-learning anomaly detectors and two simpler screens, the index selects a different top cohort: doctors whose spending is only moderately raised, but whose new-case and therapy-change counts exceed three times the centre median. In an internal concordance check, two co-author reviewers blinded to the score rated 60 cases sampled partly by index rank; they agreed with each other (Cohen’s κ=0.87, 95% CI 0.70 to 1.00), and the index’s ranking against their consensus gives an AUC of 0.88 (95% CI 0.62 to 0.97). **Conclusions:** The index gives clinical-administrative reviewers a reproducible, explainable priority order consistent with senior expert judgement on the same indicators. Because no review outcomes are linked to the records and the window is a single quarter, it is supported as a prioritisation tool, not a validated predictor of inappropriate reimbursement; multi-quarter and outcome-linked evaluation are the next steps.

## 1. Introduction

Pharmaceutical reimbursement is one of the points at which public policy becomes concrete. It touches directly the patient seeking treatment, the clinician making decisions about that treatment, and the system that administers common funds. In this space a number is never only a number: it stands for a prescription, a diagnosis, a chronic patient, an acute episode, a therapy change, and a professional decision.

The way a payer reads these numbers matters for public health and for equity between patients. Reimbursed medicines are the channel through which patients with chronic disease obtain continuous treatment in tax-funded and social-insurance systems, and the reimbursement budget is a fixed envelope: what is spent on unwarranted variation in one place is not available for coverage elsewhere. At the same time, oversight that treats a high figure as suspect in itself may push prescribers toward under-treatment. How a payer decides where to look is a governance question with consequences for access to medicines, not only a technical one.

The need for a more measured reading of the data arises precisely here. A high reimbursement figure is not enough, on its own, to characterise the profile of a doctor or of a primary-care centre. It may reflect the age of the served population, the burden of chronic disease, the proportion of chronic patients in the panel, or the prescribed treatment regimens. A figure takes on meaning only when it is read in relation to the context in which it was produced and in relation to the peer group within which the doctor practises.

In any given quarter, a primary-care payer’s administrative records cover several thousand prescribers across the region it administers; a careful reading of every doctor’s profile is not feasible within the resources available to the clinical-administrative reviewers who do this work, and the cohort that can realistically be examined in any quarter is a small fraction of the whole. The question of which records most warrant a closer reading is therefore recurring, and a defensible priority order has institutional value of its own, independent of the specific findings any later documentary review may or may not produce. We take up this question in the Albanian primary-care reimbursement system, where it has a concrete administrative form.

Specifically, we propose a model for risk reading in pharmaceutical reimbursement, grounded in established theory from risk scoring, peer comparison, and outlier detection. The model reads several indicators together rather than separately, including realised reimbursement, plan attainment, prescription count, new cases, therapy changes, the financial effects of each, and the average cost per prescription. Each doctor is compared with the profile of the primary-care centre in which they practise, through the centre median and the relative deviation of the doctor’s value from the peer group. The score that results is, by construction, an *index of concentration* of indicators. It does not reduce a doctor’s work to a single number, and it does not claim to detect any specific clinical or administrative finding. It identifies the cases where several indicators appear simultaneously above the typical profile of the centre, and in that sense it supports a more informed decision about where a closer documentary reading of the records would be most useful. A high score is thus a reason to read a doctor’s file, not an estimated probability of misconduct.

The purpose of building the model in this form is to allow a fairer and more transparent reading of the figures. The patient must remain at the centre of access to treatment; the doctor must be read with respect for the clinical complexity behind any individual decision and for the dignity of the profession; the administration of public funds must remain transparent and accountable. A risk-reading model has value when it can hold these three concerns together.

In a later extension, when reimbursement records can be linked to historical documentary findings, the same framework can be moved from a *score* to an estimated *probability* of a documented finding by using methods such as logistic regression to estimate the relationship between indicator profiles and documented findings. The framework described in this paper is the structural step that makes the later extension well defined.

Current practice tends to satisfy one goal at a time. The single-indicator peer-comparison stream [1,2,3,4] applies transparent statistical rules (cumulative-sum or CUSUM tests, deciles, *z*-scores) to each prescribing indicator independently. These rules are easy to defend but ignore that a doctor sitting in the 90th percentile on three indicators is more informative than one sitting in the 99th percentile on one. The machine-learning anomaly-detection stream [5,6,7] captures the multivariate structure but is opaque to non-technical auditors. A third stream based on Benford’s Law and related distributional tests [8,9,10,11] is transparent but rests on distributional assumptions that do not always hold on small administrative units like single primary-care centres.

### 1.1. Aim

The aim of this study is to construct and evaluate a multi-indicator concentration index for the Albanian primary-care reimbursement system and to be explicit about what it can and cannot support. The evaluation asks three questions of the demonstration cohort: whether the sub-scores carry distinct information rather than one underlying quantity; whether the ranking is robust to the choice of component weights; and whether the ranking agrees with the prioritisation that senior clinical-administrative reviewers would make from the same raw indicators. Because no review-outcome labels are linked to the records, the study does not, and does not attempt to, establish predictive accuracy for inappropriate reimbursement.

### 1.2. Contribution

The index proposed here is a simple construction that combines multivariate scoring with transparent additive decomposition, a peer-relative comparator at the level of the primary-care centre, and an output form (named-component decomposition with per-component contributions) readable directly by clinical-administrative users. The score assigns each prescriber a value between 0 and 100 as the sum of nine weighted sub-scores; three classification thresholds (70, 40, 0) and a relative ranking (top 10%, next 20%, rest) translate the score into an actionable tier system. It runs on the fields of the payer’s own quarterly reimbursement report and requires no machine-learning infrastructure.

We demonstrate the framework on three months of anonymized administrative data from 335 doctors across 30 primary-care centres in the Albanian primary-care reimbursement system and examine it from two complementary angles: a comparison against five competitor methods (a per-centre percentile-flag count, a multivariate *z*-sum, and three machine-learning anomaly detectors), and a blinded cross-check against the prioritisation of two senior doctor-administrators on cases drawn from the cohort.

The rest of the paper is organised as follows. Section 2 reviews related work in physician profiling, peer-comparison screening, machine-learning anomaly detection on health claims, and Benford-style distributional tests. Section 3 describes the data, states the methodology in full (indicators, weights, ratio-to-points rule, classification thresholds, and the handling of edge cases), and sets out the evaluation design. Section 4 presents the results, including the cohort summary, a worked score decomposition, a component-independence analysis, a weight-sensitivity analysis, a comparison against five competitor screens, and the expert cross-check. Section 5 discusses the findings, the positioning relative to existing approaches, the implications for health-system governance, the limitations, and the path to a fuller probabilistic extension. Section 6 concludes.

## 2. Related Work

The construction draws on four streams of literature. Two recent systematic reviews [12,13] survey outlier-benchmarking methods across these streams; from them we borrow the median-and-percentile comparator with small-sample handling, adapted to the primary-care-centre scale.

### 2.1. Physician Profiling

Profiling physicians on multiple utilisation indicators was established in the 1990s [14], with later work on how outlier handling and episode attribution affect the profiles [15] and practical guidance on comparison-group construction [16]. Large recent studies confirm that primary-care variation is substantial and persists after case-mix adjustment [17,18]; Sutherland and Levesque [19] distinguish warranted from unwarranted variation, a distinction we adopt in interpreting the index, which flags concentration, not violation. Barrett et al. [20] use latent class analysis to identify prescribing-pattern classes; latent classes capture multivariate structure but are not interpretable in terms of individual indicators, which is why we keep the indicators as named components of an additive score.

### 2.2. Single-Indicator Peer-Comparison Alerts

OpenPrescribing [1] computes prescriber-level rates for many measures and applies CUSUM tests against peer means; Hopcroft et al. [2] and MacKenna et al. [3] extend this to hypothesis-free outlier ranking on national data, and Medicare flags outlier Part D prescribers by top-quartile position within specialty and state [4]. These screens are transparent and operate at scale but treat each indicator independently. The audit-and-feedback literature supports the operational value of peer-comparator data of this kind [21,22]; our framework adds the multi-indicator aggregation that a reviewer following up a flag would otherwise assemble by hand.

### 2.3. Machine-Learning Anomaly Detection

A larger stream applies unsupervised learning to provider- or claim-level data [5,6,7,23]. The systematic review by du Preez et al. [23] documents both the maturity of this literature and a persistent interpretability gap. Post hoc explanation layers such as SHAP and LIME [24,25] address the gap per prediction; De Meulemeester et al. [26] combine an autoencoder with SHAP on Belgian insurance data. These approaches produce a single anomaly score per record with, at best, a post hoc explanation; the index proposed here is decomposable by construction.

### 2.4. Distributional Tests

Benford-style screens [8,9,10,11] are transparent and cheap but rely on distributional regularities that hold on large, varied populations, not on a centre with fewer than thirty prescribers. At that scale, the first-digit distribution is dominated by sample-size effects rather than by any underlying mechanism.

### 2.5. Positioning

None of these streams offers, together, multivariate scoring, transparent additive decomposition, a peer-relative comparator at the centre level, and an output that clinical-administrative users can read without translation through a black-box score. The contribution of the index is therefore one of interpretability and operational feasibility inside a payer’s existing workflow, not a claim of higher detection accuracy. As we discuss in Section 5, which approach best identifies truly inappropriate reimbursement cannot be settled without linked review outcomes. No method in this literature has them for the Albanian setting, and the present study does not have them either.

## 3. Materials and Methods

This section describes the data source, the indicators and why they were chosen, the peer-relative ratio rule that maps each indicator to a five-bin sub-score, the weights, the formal definition and algorithm, the handling of edge cases, how a high score should be interpreted, and the design of the evaluation.

### 3.1. Setting and Data

We use anonymised administrative reimbursement data from the Albanian primary-care reimbursement system, accessed through the Tirana Regional Health Insurance Fund, the custodial institution for primary-care reimbursement records in the Tirana region (one of the co-authors is the Fund’s director). The analysis window is the first quarter of 2026 (January through March), which is the payer’s plan cycle. The Fund fixes a reimbursement plan target per doctor for each calendar quarter and reviews realised reimbursement against it. The dataset contains one record per (doctor, primary-care centre, quarter) for 335 prescribers across 30 primary-care centres. Centre size ranges from 1 to 37 doctors with median 4; 15 of the 30 centres have fewer than five doctors and two have more than thirty. Chronic prescriptions in the source jurisdiction follow a two-month re-issue cycle [27]; multiples of this cycle (two, four, six, and twelve months) are the natural windows for future multi-quarter analyses (Section 5).

All personal identifiers were removed before analysis. Doctor names were replaced with sequential identifiers (D001–D335) and centre names with codes (C01–C30); the mapping is held by the custodial institution and is not retained alongside the analysis dataset. Monetary values are used in the analysis but reported here only as ratios, percentiles, cohort summaries, or scores; no absolute reimbursement amount attributable to an individual doctor appears in any table or figure.

### 3.2. Indicators and Their Selection

The unit of analysis is the triple (doctor, centre, period). The framework reads only the fields the payer already collects for its quarterly reimbursement report: financial totals and case counts per doctor. It requires no patient-level clinical data. The eleven primary indicators are listed in Table 1 together with their definition, their distribution in the demonstration cohort, and the sub-score each enters; the paragraph following the table says what each reflects about prescribing.

The eleven indicators are the quantities the Fund’s quarterly reimbursement report records for each doctor; no larger candidate pool was screened, and they are the figures the Fund’s reviewers already consult when deciding which prescriber files to read. The scoring rule and weights were set by the authors, drawing on the review experience of the co-authors who direct the regional fund and teach clinical medicine. The set spans the three dimensions the source system records: financial volume (plan target and realised reimbursement), case-pattern shape (acute, chronic, new cases, therapy changes), and the financial effect of each case category.

Each indicator reads a different aspect of prescribing. Realised reimbursement and plan attainment measure financial volume in absolute and in planned terms. New cases measure the rate at which the doctor adds patients to the chronic panel, and therapy changes the frequency with which established treatment is switched; their financial effects measure what each costs. Together, these four are the case-pattern shape of the doctor’s activity. Chronic cases and chronic reimbursement make up 99.9% of prescriptions and reimbursement in this cohort, so they coincide with the prescription count and realised reimbursement and are not scored separately. Acute prescribing is the reverse. It is rare in the reimbursement scheme (63 of 335 doctors record any, and the centre median is zero in 26 of 30 centres), so an elevated acute count marks a doctor whose reimbursed activity departs from the chronic-panel pattern of peers, for instance, by reimbursing episodic treatment that peers do not, and it enters as a low-weight component with small-count handling (Section 3.6). Acute reimbursement is context only.

From the primary inputs the framework derives secondary indicators: plan-attainment percentage (realised reimbursement over plan target; cohort median 102.9%, IQR 85.2–114.1%), mean cost per prescription (realised reimbursement over prescriptions; median 2281, IQR 2045–2623 currency units), and, for context display, the weight of new cases and therapy changes in prescriptions and the mean cost per new case and per therapy change.

### 3.3. Peer-Relative Deviation

Each doctor is compared against the median of their own primary-care centre rather than against an absolute reference. The median is preferred over the mean because it is a robust location estimator with breakdown point 50%, suited to small peer groups in which the extreme value is itself the signal of interest [28]; known limitations of peer-relative comparison [29,30] are addressed by choosing the centre as the peer boundary (a defensible one in the source system, since doctors in a centre serve the same catchment) and by the small-count rule in Section 3.6.

For each indicator *X*, denote by $X_{d,c}$ the doctor’s value, by ${\tilde{X}}_{c}$ the median across all doctors in centre *c*, and by $r_{d,c}^{X}=X_{d,c}/{\tilde{X}}_{c}$ the ratio. The ratio is read on five levels: r≤1.0 (typical for the centre), 1.0<r≤1.5 (above typical profile), 1.5<r≤2.0 (noticeable deviation), 2.0<r≤3.0 (high deviation), and r>3.0 (very high deviation). Plan attainment is a percentage rather than a peer-relative ratio and reads against absolute thresholds: ≤100%, (100,110]%, (110,125]%, (125,150]%, >150%.

### 3.4. Sub-Scores and Weights

The index $I_{d}\in \left[0,100\right]$ is the sum of nine sub-scores, in the weighted-additive form of multi-criteria decision analysis [31]. Seven derive from a peer-relative ratio against the centre median, one from plan attainment, and one from the doctor’s own historical median. Each sub-score takes 0%,25%,50%,75% or 100% of its weight according to the bin into which *r* falls. The weights are given in Table 2.

The weights place 55 points on financial volume (reimbursement, plan attainment, financial effects), 30 on case-pattern shape (new cases, therapy changes), and 5 each on per-prescription cost, acute pattern, and self-history. They are stated up front so that a reader can recompute the index under alternative weights; Section 4.4 tests three alternatives.

The historical-deviation component compares the doctor’s current value to their own median across prior windows and requires at least one prior window. In the single-quarter demonstration, it is zero for every doctor, so the attainable maximum here is 95 rather than 100.

We report the score on the original scale rather than rescaling it by 100/95, for two reasons. First, the thresholds of 70 and 40 are defined on the full nine-component scale, so rescaling this quarter’s figures would make them non-comparable both with the index as defined and with any later period in which the historical component is available. Second, rescaling is a monotone transformation, so it cannot change the ranking, which is the output this paper puts forward as primary.

A reader who prefers the other convention can convert in either direction. On a rescaled 0–100 score, the present thresholds sit at 73.7 and 42.1, and applying 70 and 40 to a rescaled score is the same as applying 66.5 and 38 here. Leaving the thresholds at 70 and 40 after rescaling, rather than converting them, moves 5 of the 335 doctors between bands and takes the high band from six members to seven. Section 4.4 reports how the band counts respond to the thresholds themselves.

### 3.5. Formal Definition and Algorithm

Let D denote the set of doctors, C the set of centres, c(d) the centre of doctor *d*, and K={1,…,9} the sub-score components. For component *k*, let $X_{d}^{\left(k\right)}$ be the doctor’s value of the underlying indicator and $w_{k}$ the weight ($\sum_{k}w_{k}=100$).

For the seven peer-relative components (K∖{planattainment,historic}), the deviation ratio is

(1) $$r_{d}^{\left(k\right)}\;=\;\left\{\begin{array}{cc} \frac{X_{d}^{\left(k\right)}}{\max\;\left({\tilde{X}}_{c(d)}^{\left(k\right)},\;0.5\right)}\; & \mathrm{if}\;k=\mathrm{acute}\;\mathrm{cases},\\ \frac{X_{d}^{\left(k\right)}}{{\tilde{X}}_{c(d)}^{\left(k\right)}}\; & \mathrm{if}\;{\tilde{X}}_{c(d)}^{\left(k\right)}>0,\\ 0 & \mathrm{if}\;{\tilde{X}}_{c(d)}^{\left(k\right)}=0\;\mathrm{and}\;X_{d}^{\left(k\right)}=0,\\ \infty & \mathrm{if}\;{\tilde{X}}_{c(d)}^{\left(k\right)}=0\;\mathrm{and}\;X_{d}^{\left(k\right)}>0, \end{array}\right.$$

where ${\tilde{X}}_{c(d)}^{\left(k\right)}$ is the median of indicator *k* across the doctors in centre c(d), and the 0.5-smoothing implements the small-count rule of Section 3.6. When ${\tilde{X}}_{c(d)}^{\left(k\right)}=0$ for a non-acute component, the by-case rule of Section 3.6 applies. The plan-attainment component uses $r_{d}^{\left(k\right)}$ equal to realised reimbursement as a percentage of the plan target. The historic component uses $r_{d}^{\left(k\right)}=X_{d}^{\left(k\right)}/{\tilde{X}}_{\mathrm{hist},d}^{\left(k\right)}$, with the denominator being the doctor’s own median across prior windows.

The value function $\phi_{k}:R_{\geq 0}\to \left\{0,25,50,75,100\right\}$ maps the ratio to a percentage of the component’s weight:

(2) $$\phi_{k}\left(r\right)\;=\;\left\{\begin{array}{cc} 0 & r\leq \tau_{1}^{\left(k\right)}\\ 25 & \tau_{1}^{\left(k\right)}<r\leq \tau_{2}^{\left(k\right)}\\ 50 & \tau_{2}^{\left(k\right)}<r\leq \tau_{3}^{\left(k\right)}\\ 75 & \tau_{3}^{\left(k\right)}<r\leq \tau_{4}^{\left(k\right)}\\ 100 & r>\tau_{4}^{\left(k\right)} \end{array}\right.$$

with τ=(1.0,1.5,2.0,3.0) for the ratio-based components and (100,110,125,150) for plan attainment.

The index is the weighted sum

(3) $$I_{d}\;=\;\sum_{k\in K}\frac{w_{k}}{100}\cdot \phi_{k}\left(r_{d}^{\left(k\right)}\right),$$

so each component’s contribution $\frac{w_{k}}{100}\cdot \phi_{k}\left(r_{d}^{\left(k\right)}\right)$ is available to a reader without further computation. The classification operator is

(4) $$B\left(I\right)\;=\;\left\{\begin{array}{cc} \mathrm{high} & I\geq 70\\ \mathrm{medium} & 40\leq I<70\\ \mathrm{typical} & I<40, \end{array}\right.$$

applied independently of a relative ranking that partitions D by *I*-quantile into top 10%, next 20%, and the rest. Algorithm 1 gives the direct implementation.
**Algorithm 1** Compute the multi-indicator concentration index.**Input:** For each d∈D, the primary indicators of Table 1 and centre assignment c(d); weight vector w summing to 100.**Output:** Index $I_{d}$, classification $B(I_{d})$, and sub-score decomposition ${\left\{\frac{w_{k}}{100}\cdot \phi_{k}\left(r_{d}^{\left(k\right)}\right)\right\}}_{k}$ for each *d*.1:For each centre c∈C and component *k*, compute the within-centre median ${\tilde{X}}_{c}^{\left(k\right)}$.2:For each doctor *d* and component *k*, compute the ratio $r_{d}^{\left(k\right)}$ by Equation (1).3:For each (d,k), apply the value function Equation (2) to obtain $\phi_{k}\left(r_{d}^{\left(k\right)}\right)$.4:Sum the weighted sub-scores by Equation (3) to obtain $I_{d}$.5:Apply B from Equation (4) to classify each doctor; rank by $I_{d}$ for the relative ranking.

The pipeline is implemented in Python 3.11 with pandas 2.3 and numpy 1.26, is linear in the number of doctors and components, completes on the 335-doctor cohort in under five seconds on commodity hardware, and emits two tables: doctor-level scores with the sub-score decomposition, and centre-level medians and 75th/90th percentiles for diagnostic display.

### 3.6. Edge-Case Handling

Three cases require special handling. First, the historical-deviation sub-score is set to zero when only a single window is available, as noted above. Second, the acute-cases indicator is a small discrete count with a zero median in 26 of the 30 centres, so the plain ratio is undefined for any doctor with at least one acute case, and assigning full points whenever the median is zero would overstate the deviation. We use the smoothed denominator $\max({\tilde{X}}_{c}^{\mathrm{acute}},0.5)$ of Equation (1), which gives a single acute case in a zero-median centre a ratio of 2 (the 50%-of-weight bin) and coincides with the plain ratio wherever the median is positive. Third, for the other peer-relative indicators, a zero centre median is read by case: a doctor whose own value is also zero is typical (lowest bin); a doctor with a positive value is at maximum deviation (highest bin). This occurs for the small case-count indicators at low-volume centres, and the asymmetric reading reflects that a positive value against a peer group of zeros is informative, whereas a single acute case is not.

### 3.7. Interpretation of a High Score

The index does not assert violation. It identifies prescribers whose indicators are concentrated above the centre profile and whose records therefore warrant a closer documentary reading. A high score can arise for reasons that have nothing to do with inappropriate prescribing. Consider a doctor whose panel, within a centre, contains a disproportionate share of older patients with several chronic conditions, or who receives patients referred back from specialist care with newly initiated or changed therapies. That doctor will record more new cases and therapy changes than the centre median, a higher realised reimbursement, and possibly a higher cost per prescription, and they will score high on five or six components at once. The documentary reading of that doctor’s file would then confirm that the pattern is explained by the panel, and the correct outcome of the screen is exactly that: a file read and closed. The index has done its job by putting the file at the top of the list; it cannot, and is not designed to, distinguish this doctor from one whose elevation has no such explanation.

### 3.8. Evaluation Design

We evaluate the index as a measurement instrument [32,33]. Face and content validity are argued rather than tested. The sub-scores are indicators reviewers recognise, spanning the dimensions the source system records. Four analyses address the remaining questions. *Component independence*: pairwise correlations and principal-component analysis of the eight active sub-score bin fractions (the eight non-historic components) across the cohort. *Weight sensitivity*: Spearman rank correlation and top-*K* overlap under three alternative weight schemes (uniform; financial-heavy; case-mix-heavy), each summing to the same effective maximum of 95. *Comparison with competitor screens*: five methods run on the same data: a per-centre percentile-flag count (the number of the eight active indicators, 0 to 8, on which the doctor is in the top decile of their centre) and a multivariate *z*-sum (per-centre *z*-scores of the same eight indicators, summed) drawn from the peer-comparison stream, and Isolation Forest [34], Local Outlier Factor [35], and One-Class SVM [36] from the anomaly-detection stream, all on the same per-centre *z*-scored matrix of the eight indicators with default sklearn settings, fixed seed, and contamination (or ν) 0.1 to match the top-decile target of the percentile baseline.

Two computational details of that comparison bear stating. Per-centre *z*-scores are taken against the mean and sample standard deviation of the doctor’s own centre, where a centre holds a single doctor the standard deviation is undefined and is set to 1, giving that doctor a *z* of 0 on every indicator. In a centre of *n* doctors, the attainable values are bounded by $\left|z\right|\leq \left(n-1\right)/\sqrt{n}$, that is, 0.71 at n=2 and 1.15 at n=3 against 5.92 at n=37, so the eight doctors in the five centres with one or two doctors cannot reach |z|>0.71 on any indicator. Ties are broken by ascending anonymised doctor identifier wherever a top-*K* cutoff falls inside a group of equal scores. The rule matters because some of these scores are discrete. The index takes 59 distinct values across the cohort and the percentile-flag count only nine (0 to 8). At that screen’s top-30 cutoff, 21 doctors share a count of four flags for nine remaining places.

Finally, an *internal concordance check* compares the index ranking with the prioritisation of two senior reviewers. Two co-authors, the director of the Tirana Regional Health Insurance Fund (who routinely decides which prescriber files warrant a closer reading) and a clinical academic at the University of Elbasan, independently rated 60 cases stratified across the index range (the 30 highest-index doctors, 15 random from the medium band, 15 random from the typical band). Cases were re-labelled (R001–R060), presented in random order, and shown with the eleven indicators of Table 1 and the plan-attainment percentage, as a reviewer would normally see them; raters were blinded to the index value, tier, centre, and each other’s ratings. Each answered “Would you recommend a closer documentary reading of this doctor’s prescription records and patient notes?” (Yes/No/Unsure) with a 1–5 severity rating for Yes. We report Cohen’s κ between raters and the area under the ROC curve of the index against their consensus, under a strict endpoint (both Yes vs both No) and a soft endpoint (Unsure counted as concern), each with an approximate 95% confidence interval built from the variance estimator of Hanley and McNeil [37] and taken on the logit scale so that it cannot fall outside [0,1].

Two features of this design bound what it can establish. First, the raters are co-authors who took part in developing the framework and who rated the same indicators from which the index is built, so the analysis is a check of internal concordance and face validity, not validation against an external standard: it asks whether the index reproduces the judgement it was designed to formalise. Second, the 60 cases were selected partly by index rank, the 30 highest-scoring doctors being included by construction, so the rated set over-represents the upper index range and under-represents the typical band relative to the full cohort. The resulting AUC therefore describes discrimination within a rated set whose selection depended on the index itself and is not an estimate of how the index would perform across the full 335-doctor cohort. We return to both points in Section 5.4.

## 4. Results

### 4.1. Index Distribution and Classification

**Figure 1:**
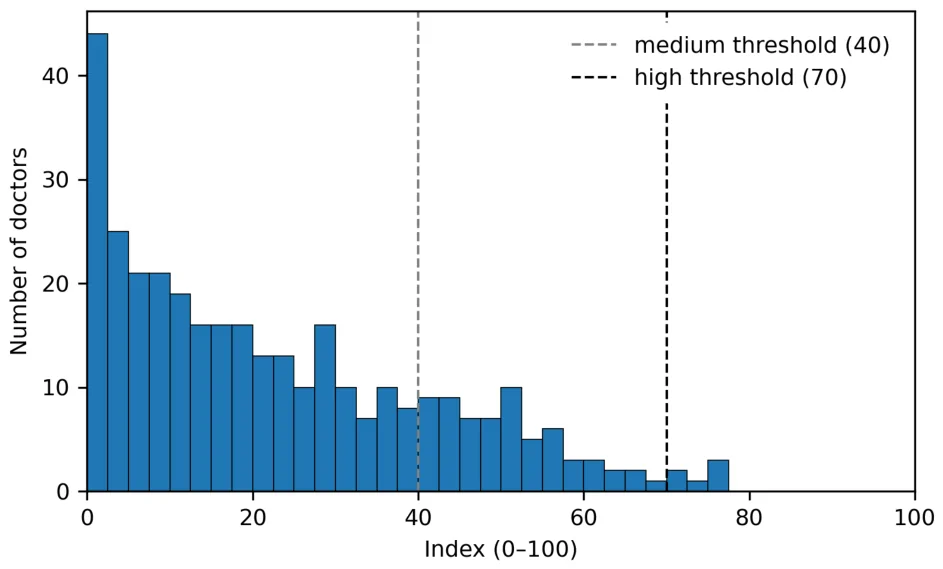
Distribution of the concentration index across the 335-doctor cohort. Dashed lines mark the medium (I=40) and high (I=70) classification thresholds. In total, 6 doctors (1.8%) sit above the high threshold, 64 (19.1%) fall in the medium band, and 265 (79.1%) remain below the medium threshold.

Across the 335 doctors, the index ranges from 0 to 76.25 (effective maximum 95), with mean 22.24, median 17.50, and 75th, 90th, and 95th percentiles of 35.00, 50.00, and 56.63. Figure 1 shows the distribution: 6 doctors (1.8%) fall in the high-concentration band (I≥70), 64 (19.1%) in the medium band, and 265 (79.1%) in the typical band. The relative-ranking partition selects the top 38 doctors (11.3%, above 10% because of ties) for the top tier, the next 67 (20.0%) for the middle tier, and the remaining 230 (68.7%) for the lower tier. The six high-band doctors are all in the top tier; these are the cases the index puts first for a closer documentary reading.

**Figure 2:**
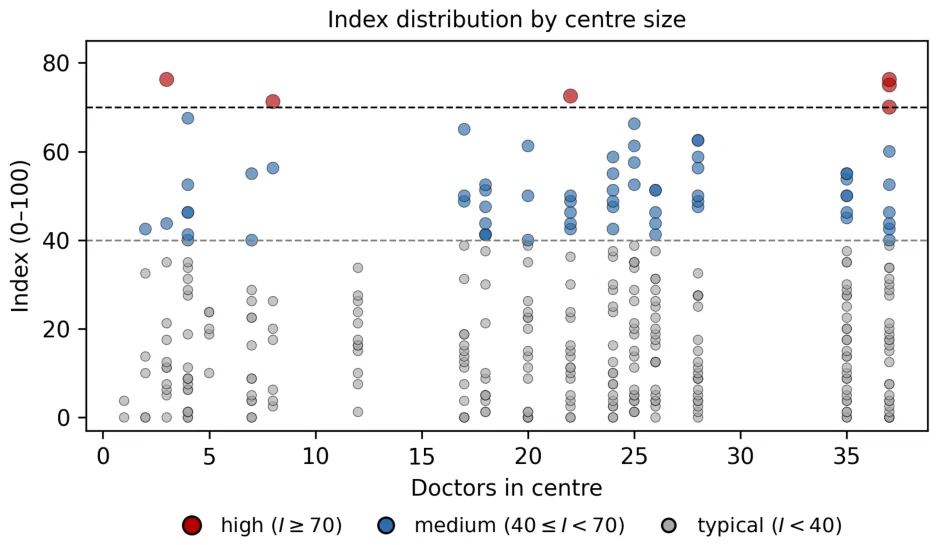
Index score against centre size. Each marker is one doctor. Dashed lines mark the medium (I=40) and high (I=70) classification thresholds. The small-centre regime (left) shows wider index dispersion, as expected when peer medians are estimated from few peers, but the high-concentration tier is not driven by small-centre noise. Of the six high-tier cases, four are in centres with at least 22 doctors, and only two are in centres with fewer than 10.

Because half the centres have fewer than five doctors, the peer median is estimated from few peers in the small-centre regime and index dispersion is correspondingly wider there (Figure 2). With only one or two peers, the median comparator is noisy, and individual doctors can produce extreme ratio values for purely structural reasons. The limiting case is a centre with a single doctor, of which there are two: that doctor is their own median, every peer-relative ratio is exactly 1, all seven ratio components score zero, and only plan attainment can contribute, giving index values of 3.75 and 0.00. The high tier is largely not an artefact of small-centre noise. Four of the six high-tier cases are in centres with at least 22 doctors and only two are in centres with fewer than 10.

Two analyses test how far the small centres carry the result. Excluding the 44 doctors in the 15 centres with fewer than 5 doctors leaves the retained ranking unchanged, which is a property of the construction rather than a finding, since the index is computed within centre, so dropping whole centres cannot alter any remaining doctor’s score. What it does alter is the composition of the priority list, and one high-band case (in a three-doctor centre) leaves the top six with it. The informative test is therefore to change the comparator instead of removing the doctors. Scoring the 44 small-centre doctors against the pooled median of all small-centre doctors, and leaving the other 291 untouched, gives ρ=0.947 against the reported index, with 22 of the top 30 and four of the top six retained; their scores move by a median of 12.50 points, 27 rising and 10 falling. The high band becomes seven doctors: two enter and the three-doctor-centre case leaves. The ordering is thus largely insensitive to the treatment of small centres, while membership of the high band at the margin is not, which is consistent with reading that band as a soft classification (Section 4.4).

Table 3 lists the ten highest-scoring doctors. All six high-band cases exceed their plan target (attainment 113.7% to 211.6%), so financial volume and multi-indicator concentration tend to co-occur. Only 4 of the 30 centres contain a high-band doctor, and 1 centre (C10) contains three of the six. Whether that reflects a centre-level practice pattern, an unusual patient mix, or three independent profiles is a question for the documentary review, not for the score. The clustering is also consistent with the multi-indicator design. Doctors flagged by the index are not simply those with the highest absolute reimbursement, which would scatter across centres in proportion to centre size, but those whose pattern deviates from their local peer reference.

### 4.2. Multi-Indicator Concentration and a Worked Decomposition

The central empirical claim is that high-concentration doctors are flagged by the simultaneous elevation of many indicators, not by one extreme value. Counting, for each doctor, how many of the eight active sub-scores are strictly positive, the median count is 7 (range 6–7) among high-band doctors, 6 (range 5–8) among medium-band doctors, and 3 (range 0–8) among typical-band doctors. The medium band is wider, five to eight active components, and includes doctors who activate many components at low bins, while the high band sits at six or seven components scoring in the upper bins. The index registers both patterns. Every one of the top-ten doctors is active on all six main components (reimbursement, plan attainment, new cases and their financial effect, therapy change and its financial effect); cost per prescription and acute cases each fire in 4 of the 10.

This pattern would not be visible to a single-indicator alert. A doctor whose reimbursement and plan attainment alone are elevated would be flagged by a CUSUM or percentile-based screen, but would not look qualitatively different from a doctor whose reimbursement, plan attainment, new cases, therapy changes, and the financial effects of both are elevated together. The index separates these two cases.

To make the decomposition concrete, doctor D292 (centre C19), one of the two highest-scoring cases at I=76.25, breaks down as shown in Table 4.

The doctor’s plan attainment of 128.8% falls in the 125–150% bin (0.75×15=11.25 points); realised reimbursement is 2.2 times the centre median (75%-of-weight bin, 15 points). The four components at maximum reflect that the median doctor in C19 recorded zero new cases and zero therapy changes during the quarter, while D292 recorded 15 new cases and nine therapy changes, so each ratio falls in the highest bin under the zero-median rule of Section 3.6. Acute cases and cost per prescription are near centre values and contribute nothing. The decomposition is the actionable artefact of the framework. It does not state that D292 has done anything wrong; it tells a reviewer which six aspects of the profile diverge from the local peer reference and which three do not so that a follow-up can request the records corresponding to the elevated components rather than the full prescription stream.

**Figure 3:**
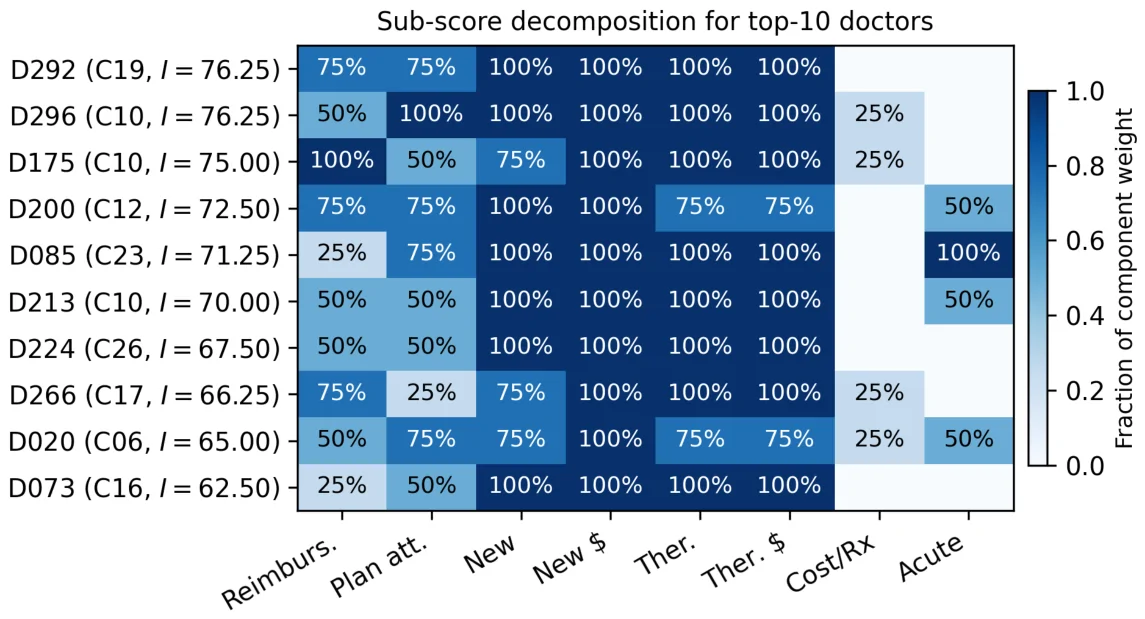
Sub-score decomposition for the ten highest-scoring doctors in the cohort. Each row is a doctor (labelled with identifier, centre, and total index); each column is one of the eight active sub-scores. Cell shading shows the fraction of that component’s weight the doctor earned. 100% is the top bin (value more than three times the centre median, or plan attainment above 150%); 75%, 50% and 25% are the intermediate bins of Section 3.5; white is zero (at or below the centre median, or plan attainment at or below 100%). Column labels: Reimburs. = reimbursement; Plan att. = plan attainment; New = new cases; New $ = financial effect of new cases; Ther. = therapy change; Ther. $ = financial effect of therapy change; Cost/Rx = mean cost per prescription; Acute = acute cases.

Figure 3 extends the decomposition to the ten highest-scoring doctors. Every top-ten doctor scores at or near maximum on the four case-pattern components; the variation in total index across the ten (62.5 to 76.25) is driven mostly by reimbursement and plan attainment; and cost per prescription and acute cases contribute sporadically and never drive the high-tier classification by themselves.

### 4.3. Independence of the Sub-Score Components

**Figure 4:**
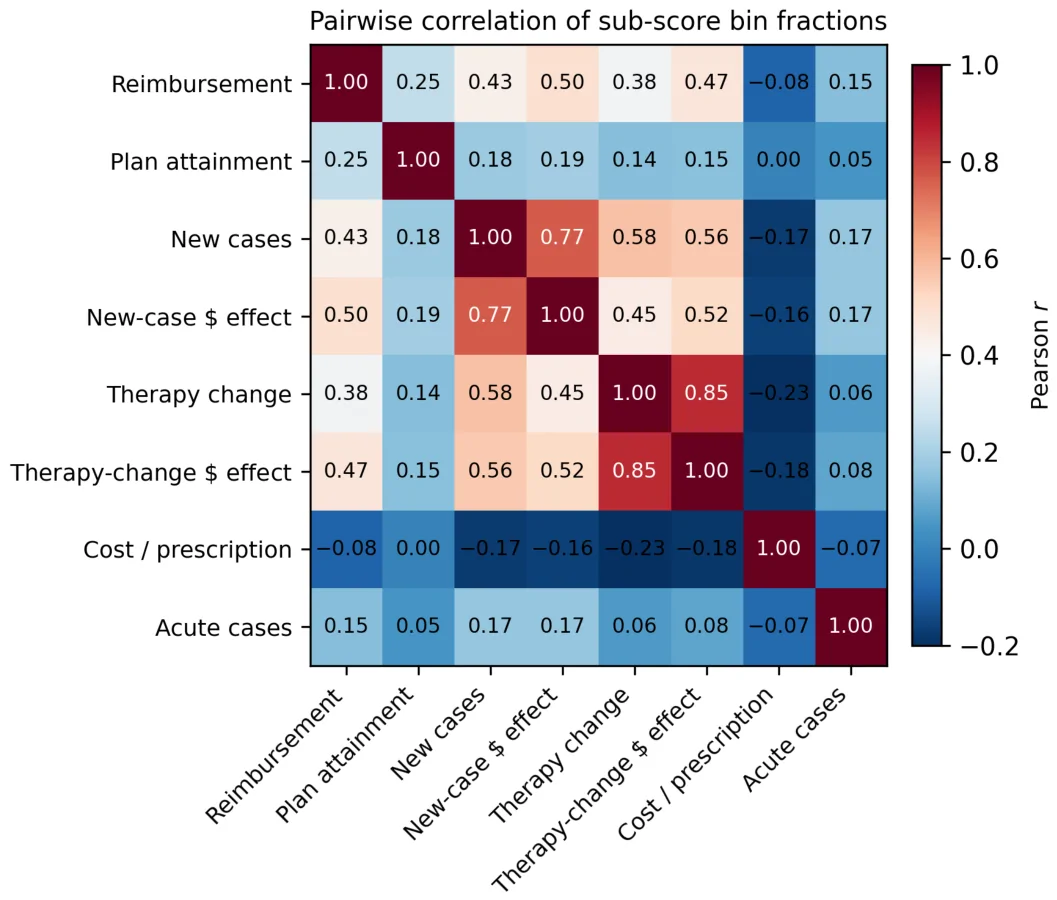
Pairwise Pearson correlation matrix of the eight active sub-score bin fractions across the 335-doctor cohort. The only two dark off-diagonal cells are the within-category pairs, in which the count and the financial effect of the same case type co-vary by construction; every cross-category pair is lighter, and the three weakly correlated components (plan attainment, cost per prescription, acute cases) appear as pale rows and columns.

If the eight active sub-scores were nearly proportional to one another, the framework would reduce to a single weighted indicator. Table 5 and Figure 4 show that they are not. The mean absolute pairwise correlation across the 28 pairs is 0.28 (median 0.18). Two pairs are strongly correlated, both within-category: therapy change and its financial effect (r=0.85) and new cases and their financial effect (r=0.77); this is expected, since the count and the financial effect of the same case category measure adjacent quantities. Reimbursement correlates moderately (r≈0.4 to 0.5) with the case-pattern components; no cross-category correlation exceeds 0.6; plan attainment, cost per prescription, and acute cases correlate weakly with everything else (|r|<0.3). Four principal components are needed to explain 80% of the variance and five to explain 90%. The eight sub-scores therefore carry meaningfully different information rather than redundantly indexing a single underlying dimension. At most two within-category pairs are collapsible, leaving six effectively independent dimensions.

### 4.4. Sensitivity to Scoring Assumptions

Table 6 summarises the agreement between each alternative weight scheme and the original. The Spearman rank correlations lie between 0.933 and 0.983, so the ordering of doctors is largely preserved. The six-doctor high band is fully preserved under uniform weighting and reduces to four of six under the two stronger reweightings, where two borderline members are replaced by doctors whose profile aligns with the emphasised component class; at the wider top-30 cutoff, 18 to 25 of the original 30 doctors are retained. For operational use, this argues for treating the relative ranking as the primary priority list and the high-tier classification as a soft cutoff sensitive to weight choice.

#### 4.4.1. Collapsing the Correlated Pairs

Section 4.3 identifies two within-category pairs that co-vary by construction: new cases with its financial effect (r=0.77) and therapy change with its financial effect (r=0.85). Weighting each member separately gives the shared part of that information two entries in the sum, so we ask what the ranking would be without the duplication. Removing the two financial-effect components and transferring their weight to the partner counts gives ρ=0.984 against the reported index; merging each pair into a single component scored as the mean of the two bin fractions, at the pooled weight, gives ρ=0.999. Both keep the effective maximum at 95. The redundancy is real but not load-bearing. The ranking remains very similar under both treatments, and the six-doctor high band is unchanged.

#### 4.4.2. Sensitivity to the Cut-Offs

The classification thresholds and the bin edges of the five-level ratio reading are set from review practice rather than fitted, so we report how far the output moves with them. Raising the high threshold from 70 to 75 reduces the high band from six doctors to three and lowering it to 65 raises it to nine; the medium band holds 64 doctors at a threshold of 40, 46 at 45 and 82 at 35. Scaling every ratio bin edge above 1.0 by factors from 0.80 to 1.25 leaves the ordering nearly unchanged (ρ between 0.992 and 0.996, with five or six of the top six retained) while moving the high band between nine doctors and three. The pattern matches the weight analysis above and points the same way. The ranking is the stable quantity, and the band boundaries are a soft classification laid over it, to be read as a convenience for reporting rather than as a categorical finding about an individual prescriber.

### 4.5. Comparison with Single-Indicator and Multivariate Baselines

The positioning of the index rests on the claim that the multi-indicator design captures patterns invisible to simpler or alternative screens. We test this by running five competitor methods on the same data and comparing which doctors each flags. Table 7 reports the agreement. The methods produce substantially different rankings. Spearman correlations against the index range from 0.062 (One-Class SVM) to 0.921 (*z*-sum), and top-6 overlap with the index’s high band is at most one of six for any baseline (zero for Local Outlier Factor); the baselines also disagree among themselves. Five doctors in the index’s top ten (D073, D213, D224, D266, D296) appear in none of the baselines’ top-ten sets. Their profiles share a shape. Three or four of the four case-pattern ratios (new cases, new-case financial effect, therapy change, therapy-change financial effect) exceed three times the centre median, while realised reimbursement stays between 1.2 and 2.2 times the median, and cost per prescription remains close to it (0.76 to 1.16). Their total spend resembles that of their peers; what departs is the composition of the activity behind it. The percentile-flag screen requires top-decile position on an individual indicator, the *z*-sum rewards extreme single-indicator values, and the three anomaly detectors surface points in sparse regions of the feature space, that is, unusual combinations rather than concurrently elevated profiles. The index places 50 of the 95 available points on the four case-pattern components, so a profile of this shape reaches the high band even when the totals are unremarkable.

Two readings follow. First, the 0.92 correlation with the *z*-sum tells the substantive story: at the level of gross ranking, several aggregation choices yield similar orderings, so what separates the index from the alternatives is not ranking quality but the *form* of the output. An Isolation Forest score of 0.58 or a One-Class SVM decision-function value of −1.2 is a single opaque number. It conveys that a record is anomalous without indicating which aspects of the prescribing profile produced the value and without a way for a clinical-administrative user to read it independently. The index decomposes into nine named components with explicit weights, each corresponding to an indicator the institutional user already understands, so a high score is readable directly: this doctor’s reimbursement, new-case count, and therapy-change count are each substantially elevated relative to peers in the same primary-care centre. This is a structural property of the index, not a parameter choice, and it is the property the institutional setting requires. Second, the comparison establishes that the index selects a different top cohort from the alternatives; it does not establish that this cohort is the more appropriate one. Which screen best identifies truly inappropriate reimbursement cannot be decided without linked review outcomes, which none of the methods has here.

### 4.6. Internal Concordance with Reviewer Judgement

The question this check addresses is whether the priority order the index produces matches the priority order that experienced clinical-administrative reviewers produce when looking at the same indicators. Because those reviewers are co-authors who helped design the framework, it is a check of internal concordance in the sense of Section 3.8 and not a comparison against an independent standard.

#### 4.6.1. Inter-Rater Agreement

The two reviewers agreed on 58 of 60 cases; the two disagreements (R038 and R052) sat at the medium-tier boundary and exchanged a No for an Unsure, never a Yes for a No. Cohen’s κ was 0.87 for the three-class rating (95% CI 0.70 to 1.00 from the large-sample variance of the observed κ, the upper limit truncated at the attainable maximum), 1.00 for the binary Yes-vs-not-Yes endpoint (both reviewers identified the same two Yes cases, R006 and R022, with the same severity 3), and 0.87 for Yes-or-Unsure vs. No, all in the “almost perfect” band [38]. The decision about which records warrant a closer reading was thus replicated across the two reviewers when the indicator set was held fixed.

#### 4.6.2. Agreement with the Index

The reviewers used the Yes label conservatively (two joint Yes cases). Against the strict consensus endpoint (n=52, 2 positives), the AUC of the index is 0.925 (95% CI 0.235 to 0.998); against the soft endpoint (n=58, 8 positives), it is 0.879 (95% CI 0.620 to 0.970). The three-level rating scale permits two defensible binary definitions of concern, so we report both. Both estimates are imprecise, the strict one especially. The soft endpoint, resting on eight positives, is the more informative of the two. It shows that the index ranks these 58 cases broadly as the reviewers did. Both intervals describe precision within the rated sample, not discrimination across the cohort, for the reason given in Section 3.8.

#### 4.6.3. Boundary Cases at the High Tier

Of the six high-band cases, reviewers said Yes to one (R022, I=72.5), Unsure to three (R060, I=76.25; R048, I=75.0; R037, I=71.25), and No to two (R024, I=76.25; R051, I=70.0). Four of six receiving Yes or Unsure is consistent with the design; the two concordant No calls show that the top of the list, while broadly aligned with reviewer judgement, is not indistinguishable from it at the margin; these two cases motivate the refinement discussed in Section 5.5. The six Unsure cases sit in the upper half of the index range (three in the high band, three in the medium band) and none in the typical band. The cases reviewers find genuinely hard to call sit between the clear priorities at the top and the clear typical profiles at the bottom, which is the property the design aims for.

#### 4.6.4. What This Check Is and Is Not

The ratings compare the index ranking with the prioritisation of two experienced senior doctor-administrators. They are not a reference standard for fraud, inappropriate reimbursement, or any other outcome concept, and the reviewers were not asked to identify such outcomes. Nor are they independent of the instrument, so what is measured is the agreement between the index and the review practice it formalises. What the check establishes is that the index produces a priority order that two reviewers, working independently and blinded to the score, broadly share at the top of the list and find ambiguous in the medium band. It does not establish, and the study cannot establish, how many of the prioritised files would yield a documented finding on review.

## 5. Discussion

### 5.1. Principal Findings

The demonstration supports five design properties. The index is decomposable. Every score breaks into nine named components with stated weights, so a reviewer reads not a number but the contribution of each component to it, and the scope of any follow-up (which documents, which prescription category) is read from the decomposition. The eight active sub-scores carry substantively distinct information (mean |r|=0.28; four principal components for 80% of the variance), so the multi-indicator structure is real rather than a proxy for one quantity. The ordering is robust to the weight choice (ρ between 0.93 and 0.98), to collapsing the two correlated component pairs, and to the treatment of the smallest centres, although high-band membership is a soft cutoff under reweighting and under a pooled small-centre comparator. That ordering is concordant with the prioritisation of two senior reviewers, one of whom routinely makes the very decision the index supports (AUC 0.88, 95% CI 0.62 to 0.97 on the eight-positive endpoint; κ=0.87 between raters), though those reviewers are co-authors rating the same indicators. Against five competitor screens the index selects a different top cohort, characterised by ordinary totals over strongly deviating case-pattern activity, that neither percentile-flag counting, *z*-summation, nor three anomaly detectors surface; which of these selections better identifies inappropriate reimbursement is not something the present data can settle.

### 5.2. Positioning Relative to Existing Approaches

Provider profiling, single-indicator peer alerts, machine-learning anomaly detection, and prescribing-quality assessment are established literatures, and the index does not claim to detect more than they do. Its contribution is on three other axes. Interpretability is structural rather than post hoc: the components are the indicators reviewers already read, and no second model is needed to explain a score. Operational feasibility: the index runs on the payer’s own quarterly report, on a standard scientific Python stack, in seconds, and can be recomputed each quarter by a ministry or fund unit without machine-learning infrastructure or a data-science team. Peer comparator: deviation is measured within the primary-care centre, which in this system is the natural unit of shared catchment and practice. The comparison of Section 4.5 shows that these design choices produce a different top cohort from the alternatives; it does not show that the cohort is the more appropriate one, and we do not claim it is. Establishing which screen best identifies inappropriate reimbursement requires linked review outcomes, which no method has for this setting.

### 5.3. Implications for Health-System Governance

For a public payer, the immediate value of a transparent priority order is procedural. Review capacity is allocated by a stated, reproducible rule rather than by informal impression; a doctor whose file is read can be told which components placed it on the list, in terms the doctor recognises; and the same rule applies across centres of very different size, since deviation is measured against local peers. This protects doctors in centres whose catchment is older or more chronically ill from being singled out for high absolute reimbursement; where one doctor’s panel is heavier than the centre’s, the score will be high, and the documentary reading closes the file, as in the worked example of Section 3.7. That outcome is expected and correct, and the index supports rather than replaces the professional judgement of the reviewers who read the files. Because the inputs are the fields of a routine reimbursement report, the framework transfers to other social-insurance systems that fix per-prescriber plan targets and record case categories, with the weights re-set to local review practice.

### 5.4. Limitations

Four limitations should be read alongside the results.

*The concordance check is internal, and against expert prioritisation rather than outcomes.* No documented review findings, confirmed inappropriate claims, or subsequent review results are linked to the records, so the study measures agreement between the index and experienced reviewers’ judgement about which files to read, not the accuracy with which either identifies inappropriate reimbursement. The two raters are co-authors and one directs the custodial institution; they were blinded to the score, but they helped develop the framework and rated the same indicators it is built from, so the agreement is between the index and the practice it formalises, not an external standard. Two further constraints bear on the AUC figures. First, the rated cases were sampled partly by index rank, so they form an enriched set in which the top of the index is over-represented, and the resulting values describe discrimination within that set rather than across the cohort. Second, with two positive cases under the strict endpoint and eight under the soft one, both estimates are imprecise, as the confidence intervals in Section 4.6 show. The AUC values should be read as evidence that the index sorts these cases the way senior reviewers would, and nothing more. Predictive validity is the object of the planned probabilistic extension, not of this study.

*The observation window is a single quarter.* A three-month window cannot show whether a doctor’s position is persistent or transient, cannot separate seasonal variation in utilisation from prescriber-level pattern, and cannot register drift in prescribing or the effect of policy changes such as list or reimbursement-rate revisions. The historical-deviation component is zero for every doctor for the same reason, and the index is read out of 95. Establishing stability requires recomputing the index over several consecutive quarters, and on the two-, four-, six-, and twelve-month windows aligned with the chronic-prescription cycle, and reporting how far the top tier persists across them; a cohort that persists across windows is a stronger priority list than one that moves with the slice. Both are mechanical in the existing pipeline once the data are available.

*The high-concentration threshold is a soft cutoff.* The weights, the bin edges of the ratio reading, and the thresholds of 70 and 40 all reflect the review practice of the source institution rather than a fit to documented outcomes, which no linked data currently permit. The ordering is robust across every variation we tested, namely weights, bin edges, collapsed component pairs and the treatment of small centres, with Spearman correlations at or above 0.93 throughout. Band membership is not. The high-tier exchanges two of its six members under strong reweighting, and ranges from three doctors to nine as the bin edges or the threshold itself are varied over plausible values. The absolute classification should therefore be read as a soft signal alongside the relative ranking, which is the quantity the tool is proposed on.

*Half the centres are small.* In centres with fewer than five doctors the median is estimated from few peers and individual ratios can be extreme for structural reasons; the small-count rule and the by-case zero-median rule contain but do not remove this, and in the two single-doctor centres the peer comparison degenerates entirely. The high tier in this cohort is drawn mainly from large centres, four of six from centres of at least 22 doctors, but the one high-band case from a three-doctor centre does not survive a pooled comparator (Section 4.1), which is the clearest illustration of the soft cutoff described above. In other cohorts, a specialty-stratified or pooled comparator for small centres may be needed.

### 5.5. Future Work

Three extensions follow directly. A composition-aware component that reads the share of prescriptions accounted for by new cases, therapy changes, and acute cases, separately from their peer-relative magnitudes, would distinguish a doctor whose elevation is dominated by one category from one whose elevation is spread across the panel; the two concordant No calls in the high band motivate it. Multi-window stability analysis, as described above, once at least a year of data is available. And the probabilistic extension: when documented review outcomes can be linked to the records, a logistic regression with the binary outcome as target and the nine sub-scores as predictors estimates the probability of a finding, its coefficients replace the argued weights, and the calibrated probability becomes the operational quantity. Only at that stage can the index be evaluated as a predictor rather than as a prioritisation rule.

## 6. Conclusions

We have presented a transparent, peer-relative, multi-indicator index that ranks primary-care prescribers by the degree to which several reimbursement indicators are simultaneously elevated relative to their own primary-care centre, and that decomposes every score into named components a clinical-administrative reviewer can read directly. On one quarter of anonymised data from 335 doctors across 30 centres, the components carry distinct information, the ranking is robust to the choice of weights and to the other scoring variations we tested, the index selects a different top cohort from five competitor screens, and its ordering is concordant with the blinded prioritisation of two senior reviewers, who are co-authors rating the same indicators.

The evidence supports the index as a prioritisation tool: a reproducible, explainable rule for deciding which prescriber files a payer with limited review capacity should read first. It does not support the index as a validated predictor of inappropriate reimbursement, and it should not be used as one. That step requires linked review outcomes and multi-quarter data, and the framework is built so that, when they become available, the same components carry over into a calibrated probabilistic model. Until then, a high score is a reason to read a file, and the reading, not the score, is where the judgement lies.

A risk-reading model of this kind has value when it holds three things together: the patient remains at the centre of access to treatment, the prescriber is read with respect for the clinical complexity and professional dignity behind any individual decision, and public funds are administered transparently and accountably. The index proposed here is designed in that spirit. It is a tool for informing how a closer documentary reading of the records should be prioritised, not for replacing the judgement of those who carry that reading out, and it should be used as such.

## Figures and Tables

**Table 1:** Primary indicators read by the framework, one record per (doctor, centre, quarter). “Median [IQR]” is across the 335 doctors of the demonstration cohort; monetary values are in thousands of currency units and are cohort summaries, not attributable to any individual. “Zero” is the number of doctors with a zero value. “Sub-score” gives the sub-score of Section 3.4 into which the indicator enters; “—” means the indicator is used for context and derived quantities only.

Indicator	Definition (Per Doctor, Per Quarter)	Median [IQR]		Zero	Sub-Score
Plan target	Reimbursement planned by the payer for the doctor	2123	[876; 3071]	1	2 (denominator)
Prescriptions	Number of reimbursed prescriptions written	987	[353; 1379]	0	7 (denominator)
Realised reimbursement	Amount actually reimbursed for the doctor’s prescriptions	2176	[835; 3149]	0	1; 2 (numerator)
Acute cases	Prescriptions for acute (single-episode, non-cyclic) conditions	0	[0; 0]	272	8
Acute reimbursement	Amount reimbursed for acute prescriptions	0	[0; 0]	272	—
Chronic cases	Prescriptions for chronic (cyclic, re-issued) conditions	987	[353; 1378]	0	—
Chronic reimbursement	Amount reimbursed for chronic prescriptions	2174	[835; 3149]	0	—
New cases	Prescriptions initiating reimbursed treatment for a patient not previously treated for the condition	17	[6; 29]	48	3
Financial effect of new cases	Reimbursement attributable to new-case prescriptions	30	[6; 59]	48	4
Therapy changes	Prescriptions replacing an existing chronic therapy with a different reimbursed therapy	11	[2; 24]	66	5
Financial effect of therapy changes	Reimbursement attributable to therapy-change prescriptions	56	[6; 116]	66	6

**Table 2:** Weight carried by each of the nine sub-scores, in points out of 100. The historical-deviation component is zero in a single quarter, so the attainable maximum within one window is 95.

#	Sub-Score	Max Points
1	Reimbursement vs. centre median	20
2	Plan attainment	15
3	New cases vs. centre median	15
4	Financial effect of new cases vs. centre median	10
5	Therapy change vs. centre median	15
6	Financial effect of therapy change vs. centre median	10
7	Mean cost per prescription vs. centre median	5
8	Acute cases vs. centre median (small-count handling)	5
9	Deviation from the doctor’s own historical median	5
	Total	100

**Table 3:** Ten highest-scoring doctors in the demonstration cohort. The ten cases come from eight distinct primary-care centres. C10 (a 37-doctor centre) contributes three cases, and each remaining centre contributes one. Plan attainment varies from 107.6% to 211.6% across the ten, indicating that the index ranks doctors by concurrent concentration across multiple components rather than by plan attainment alone.

Doctor	Centre	Index	Plan Attainment (%)	Classification
D292	C19	76.25	128.8	high
D296	C10	76.25	211.6	high
D175	C10	75.00	118.9	high
D200	C12	72.50	147.0	high
D085	C23	71.25	149.5	high
D213	C10	70.00	113.7	high
D224	C26	67.50	113.0	medium
D266	C17	66.25	107.6	medium
D020	C06	65.00	134.1	medium
D073	C16	62.50	111.2	medium

**Table 4:** Sub-score decomposition of doctor D292 of centre C19, one of the two highest-scoring doctors in the demonstration cohort. Points are shown against the maximum each sub-score can carry.

Sub-Score	Points
Reimbursement vs. centre median	15.00/20
Plan attainment	11.25/15
New cases vs. centre median	15.00/15
Financial effect of new cases	10.00/10
Therapy change vs. centre median	15.00/15
Financial effect of therapy change	10.00/10
Mean cost per prescription	0.00/5
Acute cases	0.00/5
Historical deviation	0.00/5
Total	76.25/95

**Table 5:** Pairwise correlation and principal-component summary for the eight active sub-score components.

Statistic	Value
Mean pairwise |r| (28 pairs)	0.284
Median pairwise |r|	0.179
Maximum pairwise |r|	0.849
PCs needed for 80% of variance	4 of 8
PCs needed for 90% of variance	5 of 8

**Table 6:** Sensitivity of the index to alternative weight schemes, each measured against the original 20–15–15–10–15–10–5–5 partition. *Uniform*: all eight active sub-scores at 11.875 points; *Financial-heavy*: reimbursement and plan attainment raised to 40 and 25, all other components 5; *Case-mix-heavy*: new cases and therapy changes 25 each, reimbursement and plan attainment 5, financial-effect components and acute cases 10, cost per prescription 5. Spearman ρ is the rank correlation across all 335 doctors; “Top-K∩ original” is the number of the alternative scheme’s top-*K* doctors that also appear in the original top-*K*.

Weight Scheme	Spearman ρ	Top-6 ∩	Top-10 ∩	Top-30 ∩
Uniform	0.983	6/6	9/10	25/30
Financial-heavy	0.933	4/6	5/10	18/30
Case-mix-heavy	0.965	4/6	8/10	24/30

**Table 7:** Agreement between the multi-indicator index and five competitor screens on the same 335-doctor cohort. Spearman ρ is the rank correlation across the full cohort. “Top-K∩ index” counts how many of the baseline’s top-*K* doctors also appear in the index’s top-*K*. No baseline overlaps the index’s six-case high band by more than one doctor. The multivariate *z*-sum has the highest overall correlation with the index (ρ=0.92) but still only one shared case in the top six. The near-zero ρ for One-Class SVM reflects that its decision function flags points in sparse regions of the indicator space, a structurally different signal from multi-indicator concentration above peer medians. Ties are broken as described in Section 3.8. Only the percentile-flag count ties across a cutoff to a degree that affects a reported figure. Its top-30 overlap is 12 under that rule and ranges from 8 to 16 across admissible tie breaks, since 21 doctors share the cut value. The other entries are unaffected.

Baseline	Spearman ρ	Top-6 ∩	Top-10 ∩	Top-30 ∩
Per-centre percentile-flag count	0.527	1/6	2/10	12/30
Multivariate *z*-sum	0.921	1/6	3/10	20/30
Isolation Forest	0.356	1/6	2/10	12/30
Local Outlier Factor	0.294	0/6	0/10	8/30
One-Class SVM	0.062	1/6	1/10	9/30

## Data Availability

The anonymised doctor-level and centre-level summary datasets used in the analysis, together with the Python pipeline that reproduces the analyses, figures, and tables, are available from the corresponding author on reasonable request, subject to the data-access conditions of the Tirana Regional Health Insurance Fund.
